# Search Trends for Skin Cancers: Associations of Basal Cell Carcinoma, Squamous Cell Carcinoma, and Melanoma With Socioeconomic Indicators, Geography, and Awareness Campaigns

**DOI:** 10.7759/cureus.99799

**Published:** 2025-12-21

**Authors:** Berna Solak, Meryem S Sepetci

**Affiliations:** 1 Dermatology, Sakarya University Faculty of Medicine, Sakarya, TUR; 2 Dermatology, Sakarya Training and Research Hospital, Sakarya, TUR

**Keywords:** basal cell carcinoma, google search trends, melanoma, skin cancer awareness, socioeconomic development, squamous cell carcinoma

## Abstract

Background: Skin cancer incidence is rising globally. This study assessed search behavior for basal cell carcinoma (BCC), squamous cell carcinoma (SCC), and melanoma in Türkiye using Google® Trends.

Methods: We analyzed search volume index (SVI) data from Google® Trends and examined associations with education, Internet access, geography, seasonality, and socioeconomic indicators.

Results: BCC had the highest search volume, followed by melanoma and SCC. SCC was the only cancer with a significant upward trend (p<0.001). No seasonal or monthly variation was observed. BCC search volumes correlated with gross domestic product (GDP) (p=0.038) and socioeconomic development levels (SDL) (p=0.018). SCC and melanoma searches also correlated with SDL (p=0.003, p=0.017). SCC searches further increased with high school (p=0.017) and university graduation rates (p=0.003) but showed a negative association with annual ultraviolet (UV) radiation (p=0.002). No significant associations with UV exposure or education were observed for BCC or melanoma.

Conclusions: Skin cancer search volumes in Türkiye are shaped primarily by socioeconomic development, education, and digital access. The inverse SCC-UV association suggests a gap between biological risk and public awareness. Awareness campaigns should be regionally tailored to enhance engagement and prevention strategies.

## Introduction

The incidence of skin cancers, one of the most common cancers globally, has been increasing in recent years. This rise is thought to be primarily associated with increased exposure to ultraviolet (UV) radiation (due to ozone layer depletion and greater outdoor activities), an aging population, higher rates of skin biopsies, improved diagnostic accuracy leading to earlier detection, and heightened health awareness among patients [1-3].

Skin cancers pose a significant global health issue and impose a substantial financial burden. To raise awareness, various organizations have initiated public health campaigns. Evaluating the effectiveness of these campaigns and assessing patients' interest in skin cancers is crucial for informed planning, efficient resource allocation, and the development of targeted interventions [1]. The Internet, particularly Google, has become a primary source for health information, and tools like Google® Trends enable the analysis of search interest over time and across regions to gauge public awareness of skin cancers. In the digital age, 'infodemiology' has emerged as a valuable tool for monitoring public health concerns, offering real-time insights into population-level disease awareness and information-seeking behavior. In this study, we aim to examine Google search volumes in Türkiye for the most common types of skin cancer, including basal cell carcinoma (BCC), squamous cell carcinoma (SCC), and melanoma, and investigate the relationship between search behaviors and factors such as socioeconomic status, education level, Internet access, regional characteristics, and the impact of awareness campaigns. To the best of our knowledge, this is the first study in Türkiye to comprehensively evaluate the relationship between skin cancer search trends and such a wide range of socioeconomic and educational determinants.

## Materials and methods

This cross-sectional study analyzed Google search volume index (SVI) data for BCC, SCC, and melanoma between January 2013 and December 2023.

The search terms used were “Bazal hücreli karsinom + BCC,” “Skuamöz hücreli karsinom + SCC,” and “Melanom + MM” in Turkish (English equivalents: Basal cell carcinoma + BCC, Squamous cell carcinoma + SCC, Melanoma + MM, respectively).

Data on high school and university graduation rates, Internet usage, household Internet access rates, median age, and gross domestic product (GDP) for the provinces in 2023 were obtained from the Turkish Statistical Institute (TÜİK). Socioeconomic development levels (SDL) data for 2017 were retrieved from the Ministry of Industry and Trade. The definition of rural areas in this study was based on the official data provided by the TÜİK and the Ministry of Industry and Trade. UV radiation dose data for the provinces in 2023 (measured in kWh/m²/year) were provided by the Turkish State Meteorological Service.

Statistics

The Google® Trends SVI scales peak search interest as 100, reflecting relative rather than absolute volumes. For provincial analyses, the 10 provinces with the highest SVI values were evaluated. Temporal changes were assessed using Spearman correlation, while linear regression tested associations with education, GDP, SDL, UV radiation, and seasonality. Correlations with Internet usage, household access, and median age were also examined. Results with a p-value of less than 0.05 were considered statistically significant. All analyses were performed using the SPSS 25.0 statistical software package (SPSS Inc., USA).

## Results

Trends in SVI for skin cancers

**Figure 1:**
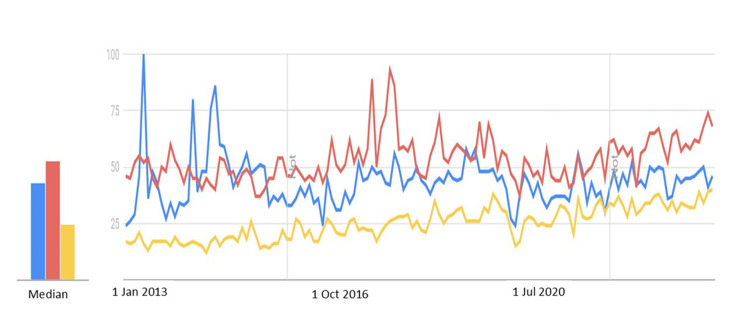
Comparison of Google® Trends search term curves: (a) Melanoma (blue), (b) BCC (red), and (c) SCC (yellow). The graph illustrates the relative interest in these search terms over the past 10 years in Türkiye. BCC: Basal cell carcinoma, SCC: Squamous cell carcinoma

**Figure 2:**
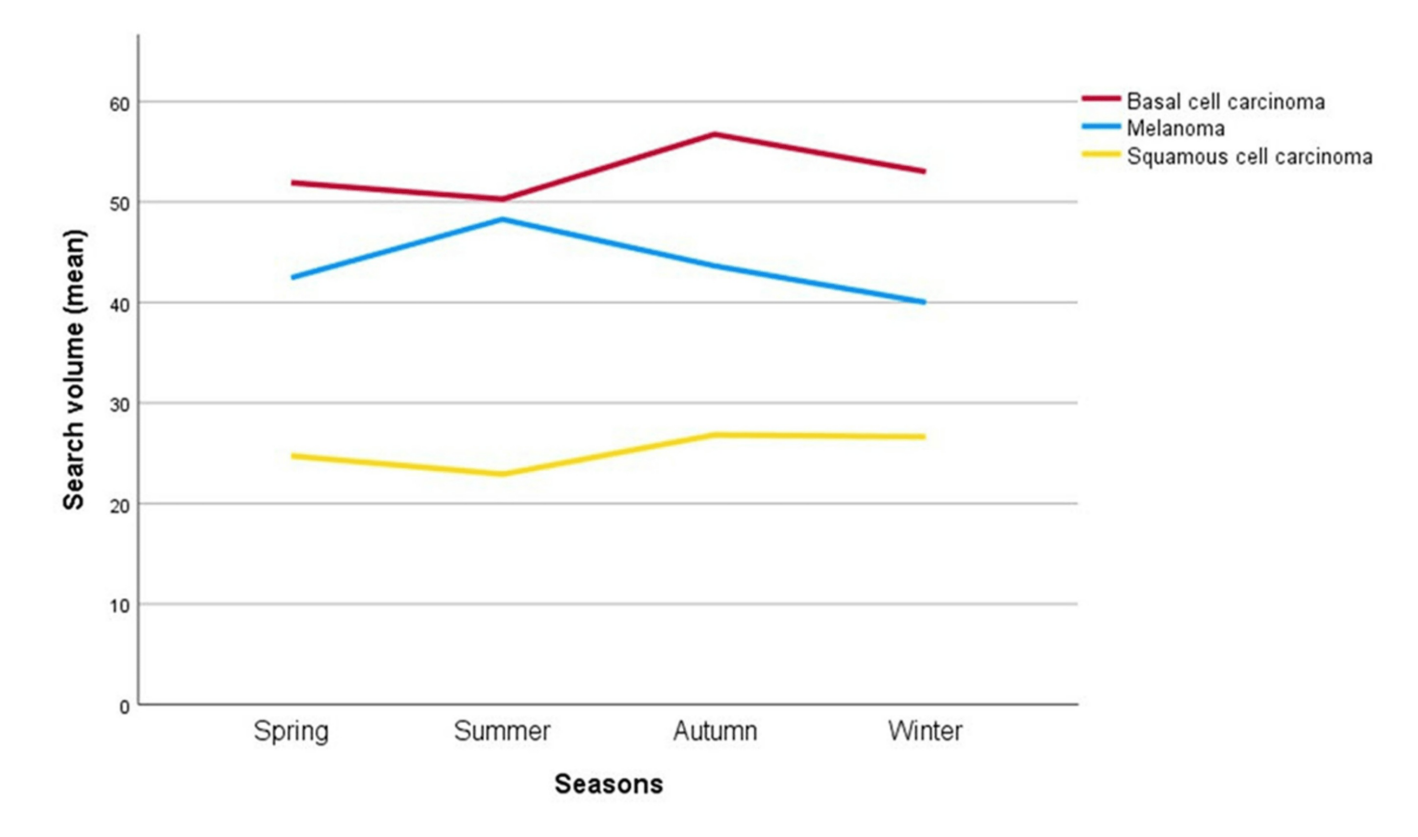
Seasonal variation of SVI for Melanoma (blue), BCC (red), and SCC (green) over a 10-year period SVI: Search volume index, BCC: Basal cell carcinoma, SCC: Squamous cell carcinoma

**Figure 3:**
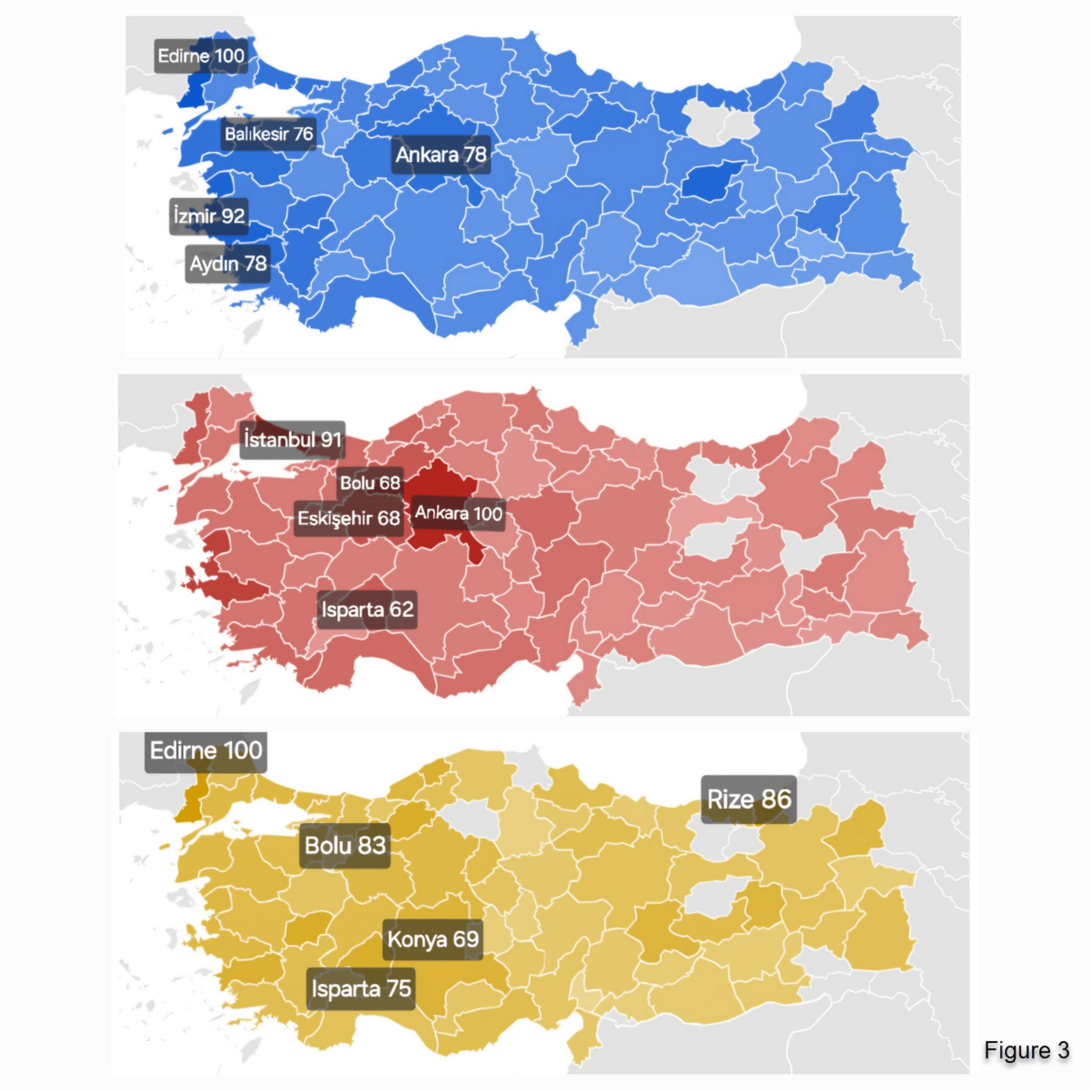
Regional interest for the search terms melanoma, BCC, and SCC across cities Darker colors represent higher SVI values. The top five cities with the highest search volumes are indicated along with their SVI values. Blue represents melanoma, red represents BCC, and yellow represents SCC. White areas on the map represent the sea, while the light-gray areas represent land. BCC: Basal cell carcinoma, SCC: Squamous cell carcinoma, SVI: Search volume index

Between 2013 and 2023, BCC consistently showed the highest average SVI, followed by melanoma and SCC, with rankings stable since 2015 (Figure 1). BCC searches rose slightly after 2017, melanoma showed modest fluctuations in 2013-14, and SCC displayed the most distinct rise in recent years. These findings suggest persistently high public interest in BCC, limited melanoma increases, and a notable SCC rise. Seasonal variations in search volume indices are illustrated in Figure 2. The intensity of SVI across provinces is presented in Figure 3. Edirne had the highest SVI for melanoma and SCC, Ankara for BCC. Edirne showed high interest across all three cancers.

SCC showed a significant long-term SVI increase (r=0.951, p<0.001), while BCC and melanoma exhibited no sustained changes despite temporary rises (p=0.154, p=0.895).

No significant seasonal variation was observed for SCC, BCC, or melanoma (p=0.324, p=0.353, p=0.236) (Figure 2). Similarly, linear regression analysis, conducted to assess the impact of May awareness campaigns, revealed no significant changes in melanoma SVI values across different months (p=0.619).

The intensity of SVI across provinces is presented in Figure 3. Edirne had the highest SVI for melanoma and SCC, Ankara for BCC. Edirne showed high interest across all three cancers.

Relationship between median age of provinces and SVI for skin cancers

No significant correlation was found between the SVI for the three skin cancers (SCC, BCC, and melanoma) and the median age of the provinces (p=0.269, p=0.269, and p=0.612, respectively).

Relationship between GDP, SDL, and SVI for skin cancers

When analyzing the 10 provinces with the highest search volumes, a significant positive correlation was found between the SVI for BCC and both the GDP (β=3.698, 95% confidence interval (CI)=0.210-7.186, p=0.038) and SDL (β=9.700, 95% CI=3.210-16.190, p=0.018) (Table 1). For SCC, a positive correlation was identified with SDL (β=21.344, 95% CI=10.284-32.404, p=0.003), but no significant relationship was found with GDP (p=0.938). Similarly, SDL had a significant effect on the SVI for Melanoma (β=25.496, 95% CI=8.351-42.641, p=0.017), whereas no significant association was observed between the SVI for melanoma and GDP (p=0.605).

**Table 1:** Mean SVI, GDP, SDL values; high-school and university graduation rates; median age; and annual UV radiation levels of the top 10 provinces with the highest search volume for BCC (Google® Trends) GDP: Gross domestic product, HSG: Percentage of high-school graduates in provinces, SDL: Socioeconomic development levels, SVI: Search volume index, UG: Percentage of university graduates in provinces, UV Radiation: Annual ultraviolet radiation dose in provinces (kWh/m²/year), BCC: Basal cell carcinoma

Provinces	SVI	GDP ($)	SDL	HSG (%)	UG (%)	Median age (years)	UV Radiation
Ankara	100	13,919	2.71	30.67	26.78	35.4	1.47
İstanbul	91	17,340	4.05	28.87	23.4	34	1.61
Eskişehir	68	11,060	1.27	31.37	22.41	37.8	1.47
Bolu	68	11,310	0.76	29.64	18.23	37.9	1.41
Isparta	62	7,580	0.56	29.75	19.76	37.5	1.61
Zonguldak	57	9,560	0.33	27.35	15.51	40.9	1.31
Edirne	68	8,910	0.53	27.39	17.48	41.1	1.31
İzmir	65	13,202	1.92	28.3	22.52	38.4	1.49
Muğla	54	11,240	1.17	27.8	27.72	34.9	1.58
Antalya	52	11,490	1.64	29.48	21.57	36.1	1.64

Association between educational level and SVI for skin cancers

Regression analysis showed a positive correlation between SCC SVI and both high school graduates (β=6.246, 95% CI=1.529-10.963, p=0.017) and university graduates (β=5.219, 95% CI=2.316-8.122, p=0.003), indicating increased SCC interest with higher education levels. No significant associations were found for BCC (p=0.262, p=0.305) or melanoma (p=0.696, p=0.562).

Association between UV radiation exposure and SVI for skin cancers

In the regression analysis, a negative association was found between the annual average UV radiation and the SVI for SCC (β=-93.659, 95% CI=-141.569 to -45.749, p=0.002). However, no significant relationship was observed between the annual UV radiation levels and the SVI for BCC or melanoma (p=0.964 and p=0.342, respectively).

The mean SVI, GDP, and SDL values; high-school and university graduation rates; median age; and annual UV radiation levels for the top 10 provinces with the highest search volume for each cancer type are presented in Tables 1, 2, and 3.

**Table 2:** Mean SVI, GDP, SDL values; high-school and university graduation rates; median age; and annual UV radiation levels of the top 10 provinces with the highest search volume for SCC (Google® Trends) GDP: Gross domestic product, HSG: Percentage of high-school graduates in provinces, SDL: Socioeconomic development levels, SVI: Search volume index, UG: Percentage of university graduates in provinces, UV Radiation: Annual ultraviolet radiation dose in provinces (kWh/m²/year), SCC: Squamous cell carcinoma

Provinces	SVI	GDP ($)	SDL	HSG (%)	UG (%)	Median age (years)	UV Radiation
Edirne	100	8,915	0.53	27.39	17.48	41.1	1.31
Rize	86	6,550	0.17	30.05	18.03	39.4	1.4
Bolu	83	11,310	0.76	29.64	18.23	37.9	1.41
Isparta	75	7,580	0.56	29.75	19.76	37.5	1.61
Konya	69	8,460	0.66	24.43	16.52	32.9	1.6
Iğdır	69	5,530	-1.17	25.61	14.99	27.6	1.48
İstanbul	66	17,340	4.05	28.87	23.4	34	1.61
Malatya	66	5,640	-0.11	28.33	18.19	35.2	1.59
Aydın	66	7,590	0.59	25.39	18.33	39.4	1.55
Kırşehir	66	7,930	-0.08	29.24	17.39	37.2	1.5

**Table 3:** Mean SVI, GDP, SDL values; high-school and university graduation rates; median age; and annual UV radiation levels of the top 10 provinces with the highest search volume for melanoma (Google® Trends) GDP: Gross domestic product, HSG: Percentage of high-school graduates in provinces, SDL: Socioeconomic development levels, SVI: Search volume index, UG: Percentage of university graduates in provinces, UV Radiation: Annual ultraviolet radiation dose in provinces (kWh/m²/year)

Provinces	SVI	GDP ($)	SDL	HSG (%)	UG (%)	Median age (years)	UV Radiation
Edirne	100	8,915	0.53	27.39	17.48	41.1	1.31
İzmir	92	13,202	1.92	28.3	22.52	38.4	1.49
Ankara	78	13,919	2.71	30.67	26.78	35.4	1.47
Aydın	78	7,600	0.59	25.39	18.33	39.4	1.55
Balıkesir	76	9,040	0.47	25.96	19.17	41.8	1.41
Tunceli	76	8,100	-0.43	32.45	21.35	39.2	1.57
Bolu	72	11,310	0.76	29.64	18.23	37.9	1.41
Çanakkale	72	11,070	0.54	26.27	20.72	41	1.37
Trabzon	72	6,170	0.38	30.17	19.91	38.1	1.39
Denizli	70	9,456	0.92	26.58	18.13	37.7	1.59

Association between household Internet access, individual Internet usage, and SVI for skin cancers

A strong correlation existed between household Internet access and individual usage across provinces (r=0.962, p<0.001). SCC SVI correlated positively with both household access (r=0.890, p<0.001) and individual usage (r=0.949, p<0.001). No significant correlations were found for BCC or melanoma with either household access (p=0.154, p=0.967) or individual usage (p=0.137, p=0.816).

Related queries of the search terms

The English translations of the top five Google® Trends queries related to “Melanoma” are: (1) "What is melanoma," (2) "Melanoma cancer," (3) "Melanoma symptoms," (4) "Skin cancer," and (5) "What is malignant." For “BCC,” the top five queries are: (1) "What is BCC," (2) "CC BCC," (3) "CC," (4) "Mail BCC," and (5) "What does BCC mean." Similarly, the top five queries for “Karsinom” are: (1) "What is carcinoma," (2) "What is squamous," (3) "What is squamous cell carcinoma," (4) "What is SCC," and (5) "What does carcinoma mean."

## Discussion

The key findings of this study indicate that among skin cancer types, BCC had the highest search volume, followed by melanoma and SCC. When examining changes over time, a significant increase in search volume was observed only for SCC, with no significant seasonal variation detected for any of the skin cancer types. Furthermore, a significant positive association was found between BCC's SVI and both GDP and SDL. In contrast, for SCC and melanoma, a positive relationship was identified only with SDL, while no significant association with GDP was observed. Additionally, the SVI for SCC was positively correlated with both high-school and university graduation rates, with interest in SCC increasing as these education levels rose; however, this relationship was not observed for BCC or melanoma. Similarly, a positive correlation was found between SCC's SVI and annual UV radiation levels, whereas this was not the case for BCC or melanoma. Finally, SCC's SVI was positively correlated with both the number of households with Internet access and individual Internet usage, a relationship not observed for BCC or melanoma.

Melanoma accounts for 1.7% of all cancers, ranking seventeenth globally, with ~330,000 new cases and 60,000 deaths reported worldwide in 2022. Incidence rates are highest in Australia and New Zealand. Although melanoma represents only ~1% of skin cancers, it causes most skin cancer-related deaths, reflecting its aggressive nature. In contrast, non-melanoma skin cancers (NMSC), primarily BCC and SCC, are far more common but often underreported in incidence statistics due to their lower aggressiveness. Approximately 744,792 new NMSC cases were recorded globally in 2022 [4]. In Türkiye, skin cancer data are available for melanoma and NMSC, though detailed BCC and SCC data remain inaccessible. Between 2009 and 2018, melanoma incidence in Türkiye increased from 1.3 to 1.9 per 100,000 in men and 1.0 to 1.5 per 100,000 in women. Similarly, NMSC incidence rose from 7.2 to 10.2 per 100,000 in men and 4.3 to 6.4 per 100,000 in women. These findings demonstrate a rising trend in Türkiye, consistent with global patterns [5].

BCC accounts for over 80% of skin cancers, which may explain its prominence in our Internet search data. Despite being more prevalent than melanoma, SCC’s lower mortality, lower recognition among the public, and higher prevalence in older individuals may explain its reduced search interest compared to melanoma. Notably, SCC was the only skin cancer in our study to show an increase in search volume over time, despite the rising incidence of all three types, raising questions for further investigation. Melanoma Awareness Month, supported by organizations such as the American Academy of Dermatology and Euromelanoma and implemented in Türkiye, aims to encourage early diagnosis and prevention. However, our study found no significant seasonal or monthly variation in melanoma search volumes, suggesting current awareness campaigns may have limited impact and underscoring the need for more engaging, targeted strategies to enhance public awareness.

Waseh et al. emphasize that large-scale cancer screening campaigns in the United States lack sufficient evidence of reducing melanoma mortality and may cause overdiagnosis and unnecessary treatments [6]. Studies show melanoma diagnoses increased after implementing such programs without a corresponding decline in mortality, suggesting these initiatives may not effectively detect lethal, advanced-stage cases. A systematic review in Saudi Arabia reported cancer screening awareness and implementation rates as low as 10-15%, emphasizing the need for community-based interventions such as education and awareness campaigns [7]. Our study reveals that there is no discernible variation in skin cancer search volumes across months or seasons in Türkiye that would correspond to campaign periods. While it is evident that Google search interest alone cannot be used to comprehensively evaluate the effectiveness of these campaigns, the findings of this study underscore a notable gap in research focused on assessing the impact of such campaigns. Thus, further studies investigating the effectiveness of awareness campaigns and screening programs appear to be necessary.

In a study examining public interest in skin cancer and melanoma terms in the United States using Google® Trends, it was found that search volumes peaked during the summer months and showed a positive correlation with melanoma mortality, which differs from our findings. However, the authors note that search volumes were not associated with melanoma incidence, suggesting that the public may not be conducting searches related to early detection. Based on these findings, they recommend that public health campaigns should be timed to focus on the period before summer [8]. A 2021 study similarly analyzed seasonal trends in public awareness of skin cancer in the United States and Australia using Google® Trends. Unlike our findings, the study revealed that searches for the terms "mole" and "skin cancer" peaked during the summer months and declined in the winter. Additionally, it was observed that searches related to artificial tanning increased before summer, while searches for sun protection terms intensified after sun exposure. These findings emphasize the importance of initiating public health campaigns prior to the summer season​ [9]. The absence of seasonal variations in our study suggests that, unlike in the United States and Australia, public awareness campaigns in our country may not effectively capture the public's attention in a seasonally significant manner. This finding highlights the need for campaigns that involve local academics, local media, and possibly celebrities, while also taking cultural differences into account.

Ahmed et al. observed that individuals with lower annual incomes are more likely to develop SCC than BCC [10]. A study from the Netherlands, examining the relationship between BCC incidence and socioeconomic status, found that the proportion of BCC patients with higher socioeconomic status has increased over time. This was attributed to greater awareness of BCC and a more proactive approach to seeking healthcare in higher socioeconomic groups [11]. Furthermore, a study from New Zealand reported a rise in the prevalence of both BCC and SCC as socioeconomic status increased, although the study did not distinguish between the two. This article explained the higher prevalence of non-melanoma skin cancer in individuals with higher socioeconomic status by increased sun exposure due to more leisure activities (such as beach outings and outdoor sports) and longer life expectancy [12]. Our study found a positive correlation between SVI for all three skin cancers and SDL, with lower volumes in less developed regions. BCC searches correlated with both GDP and SDL, while SCC and melanoma correlated only with SDL. Despite equitable healthcare access in Türkiye, low SVI in rural areas likely reflects limited education, awareness, Internet access, or trust in campaigns. SCC’s correlation with higher education underscores the role of literacy in online health-seeking. This suggests that individuals with higher educational attainment and living in socioeconomically developed regions may possess higher health literacy and better access to digital health resources, driving the increased search volume for specific medical terms. In contrast, BCC and melanoma awareness may be less education-dependent. These findings highlight the need to tailor awareness programs to rural dynamics and involve trusted local figures to improve effectiveness.

In this study, we identified a negative correlation between annual UV radiation levels and search volumes for SCC, while no significant associations were observed for BCC or melanoma. This result was somewhat unexpected, as SCC is generally considered the skin cancer most strongly linked to cumulative UV exposure. One possible explanation is that provinces with higher UV levels may also correspond to more rural or less socioeconomically developed regions, where internet penetration and health literacy are relatively limited. In such settings, online health-seeking behavior may not fully reflect the underlying biological risk. By contrast, regions with lower UV exposure but higher socioeconomic development may show greater online interest due to better awareness and access to health information.

The provinces with the highest BCC search volumes were Ankara, Istanbul, Eskişehir, Bolu, and Isparta. Ankara and Istanbul, the largest metropolitan areas, have high GDP, advanced socioeconomic levels, and dense populations, facilitating access to health information and digital platforms. For SCC, the leading provinces were Edirne, Rize, Bolu, Isparta, and Konya. For melanoma, the highest volumes were in Edirne, İzmir, Ankara, Aydın, and Balıkesir. These regions are generally more socioeconomically developed, including the capital Ankara and coastal provinces such as İzmir, Aydın, and Balıkesir, where better healthcare access and awareness activities may drive online interest.

We found a positive correlation between SCC and both "internet access" and "individual internet usage" between 2013 and 2023. Both SCC search volumes and internet access and usage steadily increased over the years, contributing to the observed correlation. The growing interest in SCC over time and its positive correlation with internet usage may suggest that digital access plays a role in shaping disease awareness. However, as correlation does not imply causation, this relationship may simply reflect the parallel upward trends of both variables over time.

In our study, for all three cancer types, the most common related queries were of the type "what is" or "what does it mean," suggesting that individuals may primarily be seeking to understand the nature of the disease. It was notable that none of the top five related queries were treatment-related. This finding could indicate that medical terms associated with skin cancers are not well-known among the general population, and that there remains a gap in basic awareness regarding what these cancers actually are.

The primary strength of this study lies in its use of a comprehensive, 11-year dataset to analyze real-time public interest in the three most common skin cancers across Türkiye. Furthermore, being the first study to correlate these search trends with detailed socioeconomic and educational indicators provides a novel perspective on the digital epidemiology of skin cancer in the region.

This study has some limitations. First, provincial-level skin cancer data in Türkiye were unavailable, restricting validation with incidence data. Second, Google Trends reflects search behavior rather than true disease burden, and results may be influenced by media coverage or terminology. Finally, while useful for gauging awareness, these findings should be complemented by registry-based studies to strengthen epidemiologic relevance.

## Conclusions

In conclusion, this study demonstrates that Internet search volumes for skin cancers in Türkiye are closely linked to socioeconomic development, education, and Internet access, while traditional risk factors such as UV exposure do not necessarily translate into greater online health-seeking activity. The negative association between SCC search interest and UV radiation suggests that regions with objectively higher biological risk may not show proportional awareness, pointing to a gap that needs to be addressed through tailored public health interventions. As the first study of its kind in Türkiye, these findings provide valuable insights for designing more effective, regionally adapted awareness campaigns and policies targeting both melanoma and NMSCs. Collaboration with local health authorities and academics will be critical to enhance the impact of these initiatives.
